# From discovery of RANKL to clinical application of anti-human RANKL antibody

**DOI:** 10.1186/ar3588

**Published:** 2012-02-09

**Authors:** Hisataka Yasuda, Yuriko Furuya, Kohji Uchida

**Affiliations:** 1 Bioindustry Division, Oriental Yeast Co Ltd, Tokyo, Japan; 2 Nagahama Institute for Biochemical Science, Oriental Yeast Co Ltd, Shiga, Japan

## 

Osteoporosis is a common bone disease characterized by reduced bone and increased risk of fracture. In postmenopausal women osteoporosis results from bone loss attributable to estrogen deficiency. Receptor activator of nuclear factor-B ligand (RANKL) is a pivotal osteoclast differentiation factor [1]. Discovery of RANKL has opened a new era in the understanding of mechanisms in osteoclast differentiation over the last decade. The discovery also results in the development of a fully human anti-RANKL neutralizing monoclonal antibody (called denosumab) and denosumab has been approved for the treatment of osteoporosis in Europe and the US.

Here I report a novel rapid bone loss model with GST-RANKL as the first topic [2]. Pharmacologic studies of candidates for the treatment of osteoporosis with this model can be done in short periods such as 3 days and a couple of weeks although it took several months in the conventional methods with ovariectomized(OVX)-rats. This model also is useful for the rapid analyses in the functions of osteoclasts in vivo. The RANKL-induced bone loss model is the simplest, fastest, and easiest of all osteoporosis models and could be a gold standard in the evaluation of novel drug candidates for osteoporosis as well as OVX.

Osteopetrosis is generally caused by failure of osteoclast-mediated resorption of skeleton. There are a numerous mouse models of osteopetrosis without osteoclasts, including c-fos deficient mice, op/op mice, RANKL-deficient mice and RANK-deficient mice. As the second topic I report a mouse model of osteopetrosis induced by a denosumab-like anti-mouse neutralizing monoclonal RANKL antibody [3]. One injection of the antibody increased bone mass markedly with remarkable decrease in osteoclast surface and number after two weeks. In addition, osteoblast surface, mineral apposition rate, and bone formation rate were also reduced markedly. These results are consistent with the recent report treating human RANKL-knock in mice with denosumab [4]. These inducible models of osteoporosis and osteopetrosis using normal mice exhibit exactly mirror images in terms of change in bone mass and are quite useful to accelerate research on osteoclast biology as well as bone metabolism in vivo.

In conclusion, the discovery of OPG/RANKL/RANK system guided us to reveal the mechanism regulating osteoclast differentiation and activation. The past decade has witnessed significant progress in the development of the RANKL antibody as a pharmaceutical agent. This is a story from a discovery of RANKL to clinical application of anti-human RANKL antibody.
